# Machine Learning Driven Channel Thickness Optimization in Dual‐Layer Oxide Thin‐Film Transistors for Advanced Electrical Performance

**DOI:** 10.1002/advs.202303589

**Published:** 2023-11-20

**Authors:** Jiho Lee, Jae Hak Lee, Chan Lee, Haeyeon Lee, Minho Jin, Jiyeon Kim, Jong Chan Shin, Eungkyu Lee, Youn Sang Kim

**Affiliations:** ^1^ Department of Applied Bioengineering, Graduate School of Convergence Science and Technology Seoul National University Gwanak‐ro 1, Gwanak‐gu Seoul 08826 Republic of Korea; ^2^ Program in Nano Science and Technology Graduate School of Convergence Science and Technology Seoul National University Gwanak‐ro 1, Gwanak‐gu Seoul 08826 Republic of Korea; ^3^ Samsung Display Company, Ltd. 1 Samsung‐ro, Giheung‐gu Yongin‐si Gyeonggi‐do 17113 Republic of Korea; ^4^ Department of Chemical and Biological Engineering College of Engineering Seoul National University Gwanak‐ro 1, Gwanak‐gu Seoul 08826 Republic of Korea; ^5^ Department of Electronic Engineering Kyung Hee University Yongin‐si Gyeonggi‐do 17104 Republic of Korea; ^6^ Institute of Chemical Processes College of Engineering Seoul National University Gwanak‐ro 1, Gwanak‐gu Seoul 08826 Republic of Korea; ^7^ Advanced Institutes of Convergence Technology Gwanggyo‐ro 145, Yeongtong‐gu Suwon 16229 Republic of Korea

**Keywords:** Bayesian optimization, design of experiment, dual‐layer channel, machine learning, oxide semiconductors, thin film transistors

## Abstract

Machine learning (ML) provides temporal advantage and performance improvement in practical electronic device design by adaptive learning. Herein, Bayesian optimization (BO) is successfully applied to the design of optimal dual‐layer oxide semiconductor thin film transistors (OS TFTs). This approach effectively manages the complex correlation and interdependency between two oxide semiconductor layers, resulting in the efficient design of experiment (DoE) and reducing the trial‐and‐error. Considering field effect mobility (𝜇) and threshold voltage (*V*_th_) simultaneously, the dual‐layer structure designed by the BO model allows to produce OS TFTs with remarkable electrical performance while significantly saving an amount of experimental trial (only 15 data sets are required). The optimized dual‐layer OS TFTs achieve the enhanced field effect mobility of 36.1 cm^2^ V^−1^ s^−1^ and show good stability under bias stress with negligible difference in its threshold voltage compared to conventional IGZO TFTs. Moreover, the BO algorithm is successfully customized to the individual preferences by applying the weight factors assigned to both field effect mobility (𝜇) and threshold voltage (*V*_th_).

## Graphical abstract

Bayesian optimization (BO) is successfully applied to the dual‐layer oxide thin film transistors (OS TFTs) design by managing two OS layers as inputs and field effect mobility and threshold voltage as outputs. BO produces TFTs with high field‐effect mobility (36 cm^2^ V^‐1^ s^‐1^) and a proper operation range. Also, machine learning provides valuable process optimization guidelines using a figure of merit.

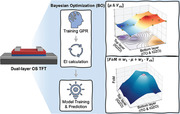

## 1 Introduction

The recent rise of advanced artificial intelligence (AI) models provides new opportunities and directions for various applications such as materials science and engineering domains. The computational power of machine learning (ML) enables it to calculate numerous possible candidates by “learning” and “problem‐solving,” leading to unprecedented innovations. The adaptive characteristics of ML has resulted in the development of many models that can be applied to various fields, including the design of experiment (DoE).^[^ ^1^ ^]^ ML‐assisted DoE provides experimenters to save time, resources, and effort by enabling automatic prediction and optimization features that take into account multiple variables and calculate feedback.^[^ ^2^ ^]^ Effective data reflection and the application of logical variable considerations alleviate the complexities inherent in variable control, streamlining the experimental process. Numerous ML‐based research endeavors have been conducted to date, including material discovery,^[^ ^3^ ^]^ decreasing the production cost of the semiconductor chip,^[^ ^4^ ^]^ optimizing thermoelectric devices,^[^ ^5^ ^]^ and predicting the bandgap structure of inorganic compounds.^[^ ^6^ ^]^ These preceding studies imply the potential and feasibility of using ML's contribution to the development of advanced science and technology.^[^ ^7^, ^8^, ^9^, ^10^ ^]^

Among many ML models, the Bayesian optimization (BO) is a model that assists the experimental design by incorporating prior knowledge to iteratively find the global optimum of a black‐box function with fewer evaluations, thereby replacing the Edisonian trial‐and‐error experimental design method typically used.^[^ ^11^, ^12^ ^]^ Gaussian process regressions (GPRs),^[^ ^13^ ^]^ a powerful tool that provides a flexible probabilistic model, were used as a surrogate model of BO to reflect the input data and derive an accurate regression. GPR allows us to not only predict the expected value of the function at unobserved points but also to estimate the uncertainty associated with these predictions. Then, expected improvement (EI) is chosen as the acquisition function to calculate the optimal recommendation using the uncertainties calculated by GPR. EI balances both exploitation (search near a targeted value) and exploration (relatively uncertain area) strategies to guide the search toward areas of maximum improvement across the entire search space. By considering both strategies, BO can recommend the most valuable subsequent experimental conditions.

The development of advanced engineering and technology raises the complexity of fabrication. Data‐driven methodology, which computes data with AI and ML, has shown greater effectiveness in complex processes such as engineering electronic devices, leading to more innovative technological development in the future.^[^ ^14^ ^]^ Most processes to fabricate electronic devices manage numerous entangled physical and chemical interactions such as plasma operation, etching, and deposition.^[^ ^15^, ^16^, ^17^ ^]^ Leveraging ML in the realm of transistor design has gained significant attention as it offers a dramatic increase in efficiency to manage complex design processes and enhances the performance of electronic devices, thereby reducing production costs.

The oxide semiconductor thin film transistor, which is emerging as a next‐generation electronic device with many advantages, has been intensively studied to improve electrical performance.^[^ ^18^, ^19^, ^20^, ^21^ ^]^ Among several studies, a dual‐layer structure has been raised for the purpose of improving field effect mobility,^[^ ^22^, ^23^, ^24^ ^]^ but it faces challenges due to the complexity of the correlation between two oxide semiconductor layers. To solve these difficulties, research on new mechanisms such as 2D electron gas,^[^ ^25^, ^26^, ^27^, ^28^ ^]^ novel oxide materials,^[^ ^29^, ^30^ ^]^ and oxide semiconductor deposition processes like homojunction have been conducted.^[^ ^31^ ^]^ However, these preceding studies demand a lot of labor and time because they require various experiments and analysis data, and sometimes go through trial and error based on human intuition. Therefore, the appropriate modification and application of the ML algorithm to optimize the dual‐layer OS TFTs provide significant time and cost advantages in DoE, compared to the traditional experimental method.

Herein, we suggest an ML‐driven design approach for engineering the higher‐level electrical performance of dual‐layer OS TFTs. To reduce the number of experimental trials required, we utilized the Bayesian optimization (BO) with Gaussian process regression (GPR) to design the dual‐layer OS TFTs. The bottom layer and top layer were considered together in the design process to show enhanced electrical performance. Specifically, the bottom layer was co‐sputtered by indium tin oxide (ITO) and indium gallium zinc oxide (IGZO) targets, while the top layer was solely deposited using the IGZO target. The entire channel layer deposition process was executed without vacuum break on the Si/SiO_2_ substrate. This procedure leads to improved throughput and guarantees high‐quality films. The main achievements can be summarized as follows: 1) By utilizing the BO algorithm to design the thickness of the dual‐layer TFTs, we fabricated devices with high field‐effect mobility of up to 36 cm^2^V^−1^ s^−1^, while simultaneously preserving other electrical performance, including stability. 2) We set the thickness of each layer as input parameters and used both field effect mobility (𝜇) and threshold voltage (*V*_th_) as output parameters since they are directly related to the device's resolution and power consumption. 3) A regression method using Figure of Merit (FoM), which can be adjusted to achieve the required performance criteria for field effect mobility (𝜇) and threshold voltage (*V*_th_), is introduced. This feasible ML‐assisted design of experiment, demonstrated by the optimization of dual‐layer OS TFTs, holds immense promise for designing complex TFT structures in the future.

## 2 Results and Discussion

### 2.1 Design Strategy with Machine Learning

ML was implemented in the design process of dual‐layer OS TFTs to engineer advanced electrical performance by determining the optimal combination of variables with minimal experimental trials. The BO method, which is an emerging ML algorithm used to identify the optimal value of the black‐box function, was applied to optimize the dual‐layer structure. An advantageous feature of BO compared to other models is that it utilizes uncertainty caused by the small volume of data for aiding experimental decisions. Therefore, BO is gaining attention as a suitable model for optimizing actual experiments.

**Figure 1:**
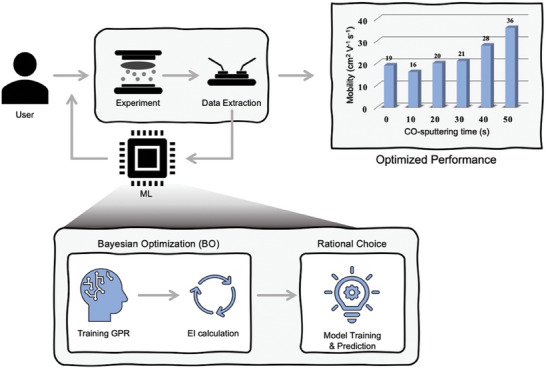
Schematic diagram of engineering dual‐layer OS TFTs. The whole process contains three steps: 1) TFT fabrication and measurement for data construction. 2) Train GPR and extract subsequent conditions by expected improvement (EI). 3) Record new data and repeat advanced steps.

The BO method employed GPR as a surrogate model to emulate unknown functions, including experimental conditions (input data) and electrical performances (output data), with expected improvement (EI) being computed from the surrogate model to suggest valuable subsequent experiments. **Figure** **1** illustrates the schematic diagram of utilizing recommended data from the ML algorithm to achieve target performance. DoE using BO is as follows: 1) TFT fabrication is conducted according to specific experimental conditions, and the electrical performance is measured to create a data set, 2) A GPR model is trained to recommend the sequential experiments, computed using EI, 3) Reiterate mentioned process, and finally, 4) it is terminated either when the target electrical performance is observed, or the user decides to terminate it. This approach can yield an optimized output with a relatively small number of iterations.

To generate an initial dataset for training the ML algorithm, the experimental process starts with the deposition of a channel layer on the prepared substrate and then measuring the electrical performance of TFTs. Input data and output data must be carefully established within a number of process parameters and performance values. The result of optimization driven by ML may show different values as the learning direction of ML changes depending on the input data and output data.^[^ ^32^ ^]^ Improving electrical performance by utilizing two oxide semiconductors is the concept of the dual layer. In order to optimize the effect of a dual‐layer structure, the thickness of each layer was set as the input data. The thickness was modulated by controlling the sputtering time of each layer. After dual‐layer TFTs were fabricated according to the selected input data set, the electrical performance parameters were extracted including the field effect mobility, threshold voltage, on/off ratio, and subthreshold swing. The other fabrication conditions such as Si/SiO_2_ substrate, annealing temperature, source/drain electrode, and patterning methods were kept uniform for all devices. Also, the conditions for data extraction were kept constant for all data. The detailed process conditions are described in the Experimental Section.

**Figure 2:**
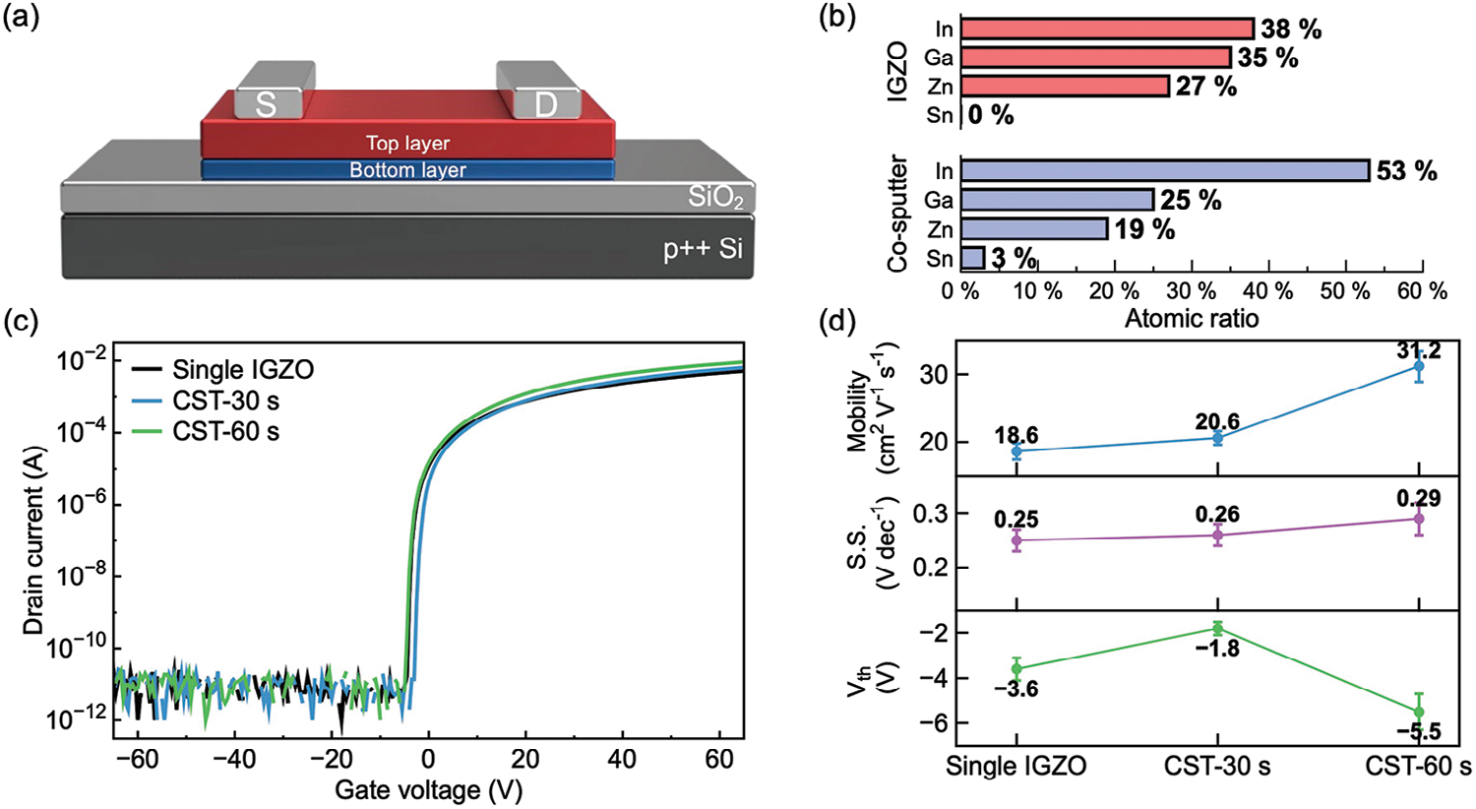
Structure and characteristics of dual layer OS TFTs. a) The device schematic of dual‐layer architecture shows that the bottom layer is deposited with ITO and IGZO, while the top layer consists of IGZO. b) The atomic ratio of single IGZO film and co‐sputter (ITO & IGZO) film. c) Transfer curves (I_DS_‐V_GS_) of three conditions (single IGZO, CST‐30 s, and CST‐60 s TFT). d) Electrical performance parameters (field effect mobility, subthreshold‐swing, and threshold voltage) of each variation (single IGZO, CST‐30 s, CST‐60 s TFT).

**Figure** **2** shows the properties of dual‐layer OS TFTs fabricated according to the initial experimental conditions. The bottom layer of the dual‐layer OS TFTs, which was fabricated by co‐sputtering IGZO and ITO, was intended to enhance the field effect mobility, while the top layer was a single IGZO to ensure stable electrical characteristics (Figure 2a). The metal cations of a single IGZO consisted of 38% In, 35% Ga, and 27% Zn, whereas the co‐sputtered layer (IGZO and ITO) was found to be 53% In, 25% Ga, 19% Zn, and 3% Sn (Figure 2b). In metal oxide semiconductors, indium and tin are major conducting paths due to the overlap of large orbitals and increasing the field effect mobility.^[^ ^33^ ^]^ Therefore, the In‐rich and Sn‐added bottom layer is expected to show high field effect mobility characteristics. To verify the field effect mobility boosting effect of the co‐sputtering layer and obtain initial data required for machine learning, OS TFTs were fabricated under three conditions, and their electrical characteristics were measured. The co‐sputtering time (CST) with IGZO and ITO was set at 0, 30, and 60 s, respectively, followed by the deposition of a single IGZO to maintain the total sputtering time of 680 s. Each device was noted as a single IGZO, CST‐30 s, and CST‐60 s. Figure 2c,d shows the electrical characteristics for each condition. As the CST increased from 0 to 30 s and 60 s, the field effect mobility increased from 18 to 20.6 cm^2^ V^−1^ s^−1^ and 31.2 cm^2^ V^−1^ s^−1^, and the subthreshold swing values also slightly increased. On the other hand, the threshold voltage (*V*_th_) positively shifted from −3.6 to −1.8 V and then negatively shifted to −5.5 V. This result is attributed to the addition of indium and tin, along with the variation of the total trap density generated during the co‐sputtering process. As mentioned, the addition of indium contributes to the enhancement of mobility, but there is an issue reported that an excess of indium leads to a negative shift in *V*_th_.^[^ ^33^ ^]^ This is observed when comparing mobility and *V*_th_ of pristine IGZO and CST‐60 s. Concurrently, the co‐sputtering process induces damage to the thin film, thereby changing its characteristics.^[^ ^34^ ^]^ Hysteresis measurements were conducted to inspect the electrical performance of co‐sputtered TFTs (Figure S1, Supporting Information). When conducting hysteresis measurements, it was observed that the *V*_th_ shift of CST‐30 s is more evident compared to pristine IGZO, indicating a relatively higher presence of interface traps between the channel and gate insulator. The increased interface traps cause a positive *V*_th_ shift in CST‐30 s compared to pristine IGZO. These experimental results confirm that co‐sputtering ITO and IGZO is an effective method to compose the bottom layer of dual‐layer OS TFTs, which improves the field effect mobility characteristics. These results provide the initial input data for establishing the GPR model.

### 2.2 Bayesian Optimization for Dual‐Layer OS TFTs

Figure 2 demonstrates the changes in characteristics caused by adjusting the sputtering time for each thickness of the dual layer. The bottom layer was modified by regulating the co‐sputtering time of ITO and IGZO, and the top layer was controlled by the sputtering time of IGZO. To achieve advanced electrical performance through engineering the dual‐layer structure, a DoE using the BO algorithm was implemented. The deposition time of the bottom layer was varied to establish input data, with field effect mobility being set as the output variable. It can be observed from Figure S2 (Supporting Information) that the device properties deteriorate rapidly beyond a co‐sputtering time of 90 s. Therefore, the optimization process was carried out by adjusting the input variable within the range of 0–80 s. The initial step in training the GPR model involved establishing the input and output data from Figure 2. To obtain noticeable variation in thickness over time, expected improvement (EI) was calculated by setting the input data at 10 s intervals (Figure S3, Supporting Information). This proper establishment of data and ML enables efficient investigation of the parameter space and identification of the most influential parameters on the output variable.

**Figure 3:**
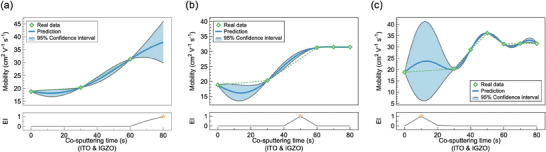
Data visualization of BO process that trains Gaussian process regression (GPR) surrogate model to predict the field effect mobility and EI of each condition of the bottom layer. Green diamond markers and dotted lines indicate the trend curve of real data. The blue solid line indicates the field effect mobility prediction for all time values between 0 and 80 s. The 95% confidence deviations are marked as light blue shadows. Star point which has maximum EI value indicates the next rational experimental time. a) Initial GP model and EI based on three data points. b) Trained GP model and EI after inputting two additional data points (80 and 70 s) based on BO proposal of (a). c) Trained GP model and EI after inputting two additional data points (50 and 40 s) based on BO proposal of (b).

The BO process was executed successfully, as shown in **Figure** **3**. As the initial data shows a consistent upward trend, BO indicates the highest EI value at 80 s, which is recommended as the most useful condition for the subsequent experiment. (Figure 3a) The recommended input variable is generated iteratively using data from previous experiments. The change in the regression shape and the corresponding shift in the highest EI point (star) was depicted. After training three experimental conditions (0, 30, and 60 s) in Figure 3a, BO recommended 80 s as the fourth experimental condition. Subsequently, Figure 3b shows the outcome after training in the fifth experimental condition, which was suggested based on the training up to the fourth experimental condition. Figure 3c demonstrates BO's recommendation of the eighth experimental condition after training on data up to the seventh experiment. And the full detail of this work process is demonstrated in Movie S1 (Supporting Information). As the input variable with a value over 50 s is learned, the uncertainty of the GPR prediction converges closer to the experimental measurement uncertainty. In addition, Figure 3c shows that there exists a region where the trend line for field effect mobility forms a maximum peak as the dual‐layer effect is optimized. The interval between 0 and 20 s exhibits an increase in uncertainty due to the lack of training data compared to other regions. This result shows that BO was effectively adjusted to attain the highest field effect mobility value by deliberating solely on the input parameter of the co‐sputtering time of ITO and IGZO. To consider the experimental distribution, the median of five values from each device under identical recommended conditions was trained. All the experimental conditions used in Figure 3 are listed in Table S1 (Supporting Information).

However, considering only one performance parameter in DoE results in the deterioration of the other device's electrical performance. Each performance parameter is correlated, including the field effect mobility (μ), threshold voltage (*V*_th_), subthreshold swing (S.S.), and the on/off ratio. Even if mobility increases, the TFT cannot switch to the gate voltage within its operating range when *V*_th_ is extremely shifted in a negative direction. Achieving a high field effect mobility is crucial for high‐resolution displays while maintaining an appropriate *V*_th_ is necessary for minimizing the power consumption of the gate driver circuit in displays.^[^ ^35^, ^36^ ^]^ So, it is important to establish an ML algorithm that considers variables appropriately and utilizes them in the design process. For the practical design of dual‐layer OS TFTs, the BO algorithm was modified to incorporate the thickness of each layer as input variables, and field effect mobility (μ) and threshold voltage (*V*_th_) as output variables. Even from the perspective of DoE, it is more reasonable to simultaneously calculate multiple variables to achieve the desired characteristics.^[^ ^1^ ^]^

The algorithm used in Figure 3 was modified to account for the increased complex correlation among variables resulting from the adoption of a dual‐layer structure. Both the deposition times of the bottom layer and top layer were set as input variables. Since only one input variable was considered in Figure 3, GPR was trained using the radial basis function (RBF) kernel. However, as both layers affect the performance, the Rational Quadratic (RQ) kernel was used to better capture the correlation of input variables.^[^ ^37^ ^]^ The modified algorithm was trained by the data recorded in Figure 3. However, it should be noted that the total value of two input variables in Figure 3 amounted to 680 s. It only represented the diagonal area of the entire matrix which has a range of 600–680 s. To conduct a broad search of the matrix, additional data on various boundary conditions was trained for each input data.

**Figure 4:**
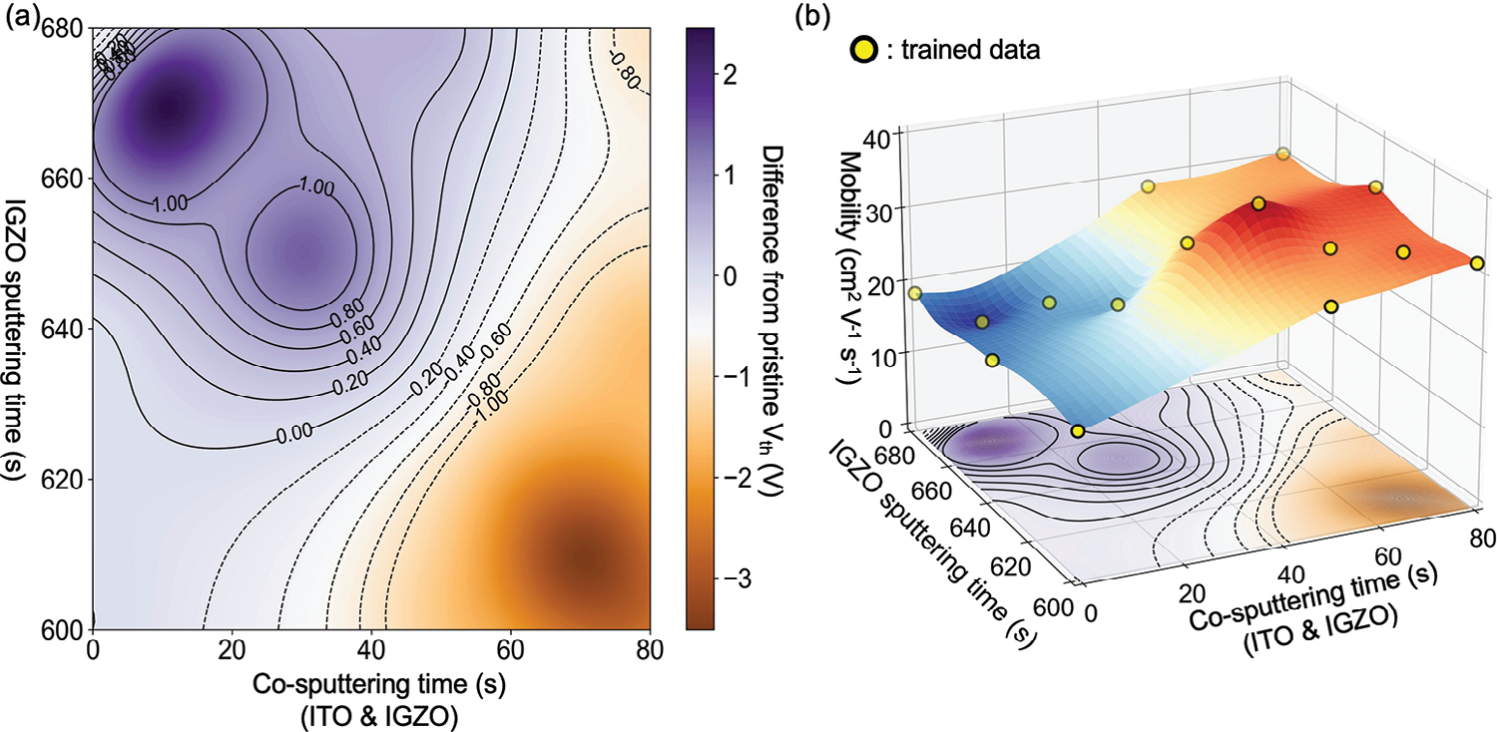
Illustration of GPR which treats two input features: Co‐sputtering time of ITO and IGZO, and IGZO sputtering time. a) The expected degree of difference from pristine *V*_th_ was shown as a GPR contour map. b) The expected μ value and the *V*_th_ difference were expressed as one 3D coordinate.

**Figure** **4** shows the GPR for two input and two output variables. To account for the *V*_th_ property of dual‐layer TFTs, a second output variable that expresses the difference of *V*_th_ compared to single IGZO TFTs was added. As depicted in Figure 4a, there exists a region where the output is close to zero. This signifies that the shift of *V*_th_ can be adjusted through the proper manufacturing of the bottom and top layers. The use of a 3D coordinate system, which expresses both field effect mobility and *V*_th_‐related outputs produced by individual trained GPR models, facilitated the prompt identification of optimal experimental conditions within a single figure. Figure 4b shows that the highest field effect mobility and the smallest change in *V*_th_ can be achieved when the CST of the bottom layer is 50 s, and the top layer is 630 s. Overall, the application of an ML‐based DoE approach successfully identified the optimal thickness combinations that fulfilled the desired properties of dual‐layer TFTs, by training two input (thickness of bottom and top) and two output (𝜇 and *V*_th_) variables, using only 15 out of 81 potential experimental trial candidates. Table S1 (Supporting Information) lists all the experimental conditions used in Figure 4, providing a detailed summary of the electrical performance used for each data point. As shown in Figure 3, a median value of five points was trained in Figure 4 to consider the experimental distribution.

To enhance the reliability of the surrogate model, additional experiments were conducted and compared with the predicted outcomes (Figure S4, Table S2, Supporting Information). Two points were selected for verification, one near the trained data and another at a relatively greater distance. Each detailed deposition time for the bottom layer and top layer were set at (40 s, 650 s) and (60 s, 670 s) respectively. The field effect mobility and threshold voltage were measured for five points and their median values are 30 cm^2^ V^−1^ s^−1^ and −1.51 V for (40 s, 650 s), and 25.7 cm^2^V^−1^ s^−1^ and −1.02 V for (60 s, 670 s). By comparing the detailed ranges of predicted values and measured values in Table S2 (Supporting Information), it is evident that GPR functions suitable as a surrogate model for BO.

**Figure 5:**
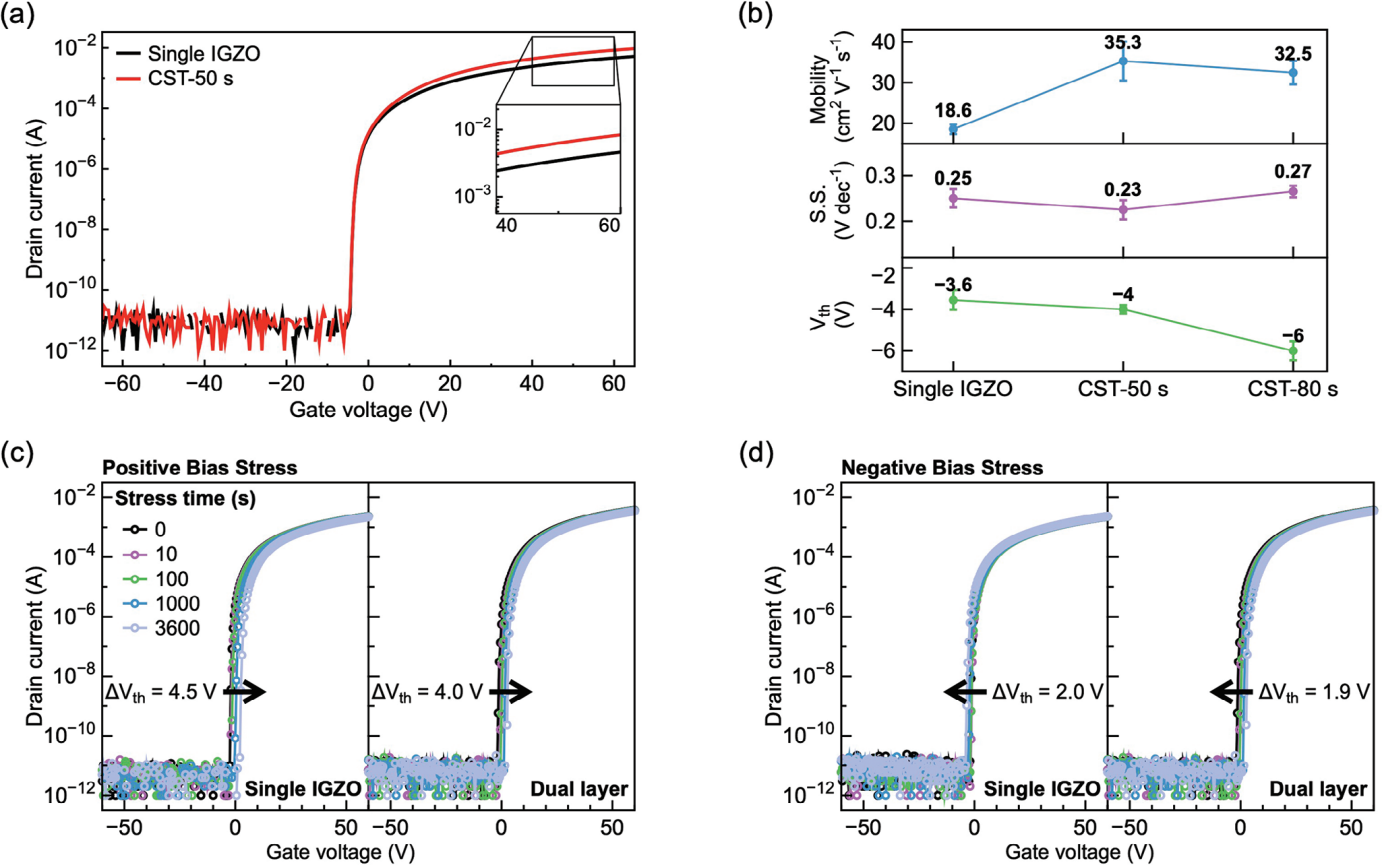
Electrical performances of the optimized device (CST‐50 s) relative to a single IGZO TFT. a) Transfer curves (I_DS_–V_GS_) of single IGZO TFT and optimized dual‐layer TFT. b) TFT electrical performance parameters (*V_t_*_h_, S.S., and μ) for single IGZO, CST‐50 s and CST‐80 s TFT. c) Positive bias stress (PBS) results were conducted at V_GS_ = 20 V and V_DS_ = 10 V from 0 to 3600 s. d) Negative bias stress (NBS) results were conducted at V_GS_ =  −20 V and V_DS_ = 10 V from 0 to 3600 s. Comparison of single IGZO and dual layer TFTs demonstrated on the left and right, respectively.

**Figure** **5** shows the electrical performance of the optimized dual‐layer TFT, which consists of a bottom layer co‐sputtered with ITO and IGZO for 50 s, and a top layer of IGZO for 630 s, as well as a single IGZO TFT for comparison. Each device was noted as a single IGZO, CST‐50 s. For additional comparison, a device with a bottom layer co‐sputtering time of 80 s and a top layer of 600 s was added, naming it CST‐80. The transfer curves for optimized TFT (CST‐50 s) and the single IGZO TFT were presented by black and red lines, respectively, and the inset indicates an increase in on‐current of the dual‐layer TFT with the similar off‐current (Figure 5a). The electrical performances depict that the field effect mobility of optimized TFT almost doubled compared to that of a single IGZO TFT (Figure 5b). The field‐effect mobility (μ) was 35.3 cm^2^ V^−1^ s^−1^ obtained by statistically analyzing ten sets of data for the optimized TFT. This field effect mobility is accomplished while maintaining a low S.S. of 0.23 V dec^−1^, with negligible difference of *V*_th_. Stability is another crucial electrical characteristic of OS TFTs, especially considering their role as switching devices. To evaluate the stability of TFTs, hysteresis was measured (Figure S1, Supporting Information). In contrast to other conditions (CST‐10 s, CST‐30 s), optimized TFT exhibited negligible hysteresis characteristics, similar to pristine IGZO. Also, positive bias stress (PBS) measurement and negative bias stress (NBS) measurement were carried out over 3600 s at *V*_DS_ of 10 V and *V*_GS_ of 20 V and −20 V respectively (Figure 5c–f). It was confirmed that the field effect mobility of dual‐layer OS TFTs was effectively improved without degradation of stability, which is consistent with a single IGZO TFT in both PBS and NBS. The optimized dual‐layer OS TFT can be directly applied and utilized without changing the electrical design rules of existing electronic circuits. Moreover, the variation of electrical characteristics with varying thickness during the optimization process coincides with the material and thickness properties reported in previous studies, as well as with the tendencies of a dual‐layer structure (Detailed explanation is discussed in Note S1, Supporting Information). This implies that Bayesian optimization is a suitable methodology for optimizing dual‐layer OS TFTs.

### 2.3 Advanced Application of Machine Learning with Figure of Merit (FoM)

Transistors require varying electronic performance standards depending on the type of electronic circuit. In high‐resolution displays, TFTs with high field‐effect mobility characteristics are essential as the available space for TFTs is restricted within one pixel or unit cell. On the other hand, the *V*_th_ is related to the power consumption of the gate driver circuit^[^ ^35^, ^36^ ^]^ of the display, which functions as a type of shift register^[^ ^38^ ^]^ because current flows even when the circuit is turned off. In general, it is difficult to optimize mobility and *V*_th_ in the desired direction simultaneously. Therefore, it is important to balance the characteristics of μ and *V*_th_ in designing electronic devices.

To overcome the challenge of designing dual‐layer OS TFTs with different standards, we replaced two output parameters (μ and *V*_th_) with the singular figure of merit (FoM). The FoM calculates a value between zero and one, based on the required engineering criteria, by assigning weight factors (*w*_1_ and *w*_2_) to each performance parameter, which sum up to one. Additionally, the performance parameter terms were normalized to a range of 0–1, following the equation:

(1) $$x_{\mathrm{normalized}}=\frac{x-x_{\min}}{x_{\max}-x_{\min}}$$

Achieving greater field effect mobility enhances electrical performance while approaching *V*_th_ closer to 0 ensures reduced power consumption. Among the data calculated by GPR, normalized mobility (μ_normalized_) becomes 1 as it reaches the maximum value and 0 as it approaches the minimum. In the case of *V*_th_, the absolute value was taken and then normalized with *x*_min_ set to 0. Subsequently, an additional normalization step was conducted to assign a value of 1 when *V*_th_ approached 0 and a value of 0 when *V*_th_ deviated further. This step ensured FoM approached a value close to 1 when the field effect mobility had a higher value and *V*_th_ approached 0, thus accurately capturing their influence on the performance. The FoM is represented by the weighted sum of the normalized field effect mobility (*m*) and the normalized *V*_th_ (*t*), where the weights *w*_1_ and *w*_2_ are assigned to each term, respectively. Detailed equations for FoM are described in Note S2 and Equations S1–S3, Supporting Information.

(2) $$FoM=w_{1}\times m+w_{2}\times t$$

**Figure 6:**
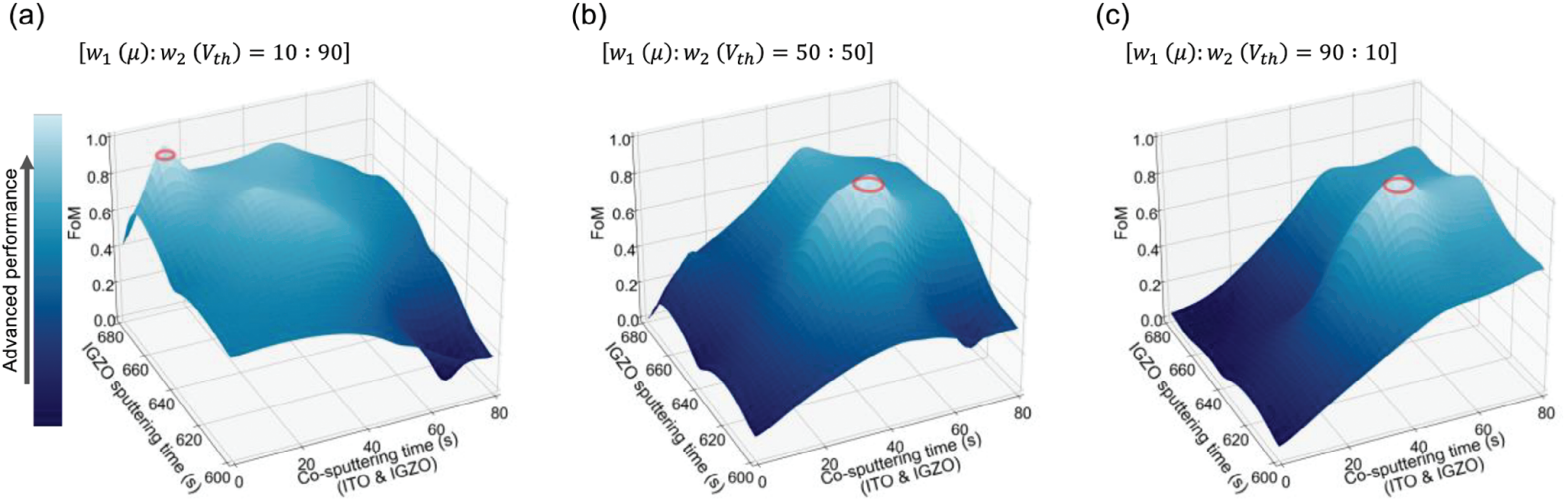
Surface plots of the figure of merit (FoM) which estimated two input features (Co‐sputtering time and IGZO sputtering time). The figure of merit was adjusted according to the variance of importance between field effect mobility (𝜇) and threshold voltage (*V*_th_). Setting weight factors (*w*_1_ and *w*_2_) assigned to μ and *V*_th_ to a) 10:90, b) 50:50, c) 90:10.

As the ML model was trained with 2 input variables (μ and *V*_th_) and derived 1 output variable (FoM) by using Equation (2), only one GPR surrogate model was computed. The GPR model using Equation (2) was executed successfully, as shown in **Figure** **6**. ML was implemented by changing the ratio of weight factor (*w*_1_ and *w*_2_) according to the criteria required for μ and *V*_th_. The FoM takes on a value close to 1 and appears white when a performance is most outstanding based on the target ratio. Conversely, the FoM takes on a value closer to 0 and appears dark blue as the performance metric decreases. In Figure 5, two output parameters were expressed in one 3D coordinate system. In contrast, Figure 6 expresses only a single parameter, which simplifies the understanding of the regression distribution. The baseline, represented by a red line in Figure 6, corresponds to a FoM of 0.95. As the GPR model calculated depending on the ratio of weight factors was changed, the surface plot of FoM and the baseline were also modified. This result demonstrates the potential for increased utilization of BO which can engineer electronic devices with desired properties by modifying the formula in a targeted approach.

## 3 Conclusion

In summary, we presented the potential for incorporating Bayesian optimization (BO) into the design process of dual‐layer OS TFTs fabricated through co‐sputtering of ITO and IGZO for the bottom layer and sputtering of IGZO for the top layer. This design strategy leads to improvement in field‐effect mobility (36.1 cm^2^ V^−1^ s^−1^) by twice that of single IGZO TFTs, while maintaining ideal electrical performance parameters such as *V*_th_, subthreshold‐swing, high on‐off current ratio, and good stability. BO also allows to require a minimal number of experimental trials which offer promising approaches to address time‐consuming and labor‐intensive experiments. Furthermore, we demonstrate the utilization of the figure of merit (FoM) by introducing a weight factor to both field effect mobility and *V*_th_, highlighting that ML can be incorporated into each device design process with simple adjustments. It implies that by leveraging FoM, ML provides a valuable process optimization guideline for feature‐centric processes. This study shows an accelerated design process for electronic devices, indicating that the appropriate utilization of advanced ML algorithms in electronic device design has promising possibilities to not only enhance performance but also save time and cost.

## 4 Experimental Section

### Fabrication of the TFTs

Single IGZO TFTs and dual‐layer OS TFTs were fabricated on heavily boron‐doped Si wafers (P^++^‐Si) that had a thermally grown layer of SiO_2_ measuring 200 nm. To prepare the wafers, they were subsequently cleaned using deionized (DI) water, acetone, and isopropyl alcohol (IPA) for 20 min each. Ultraviolet ozone (UVO) treatment was followed to remove residual impurities. Oxide semiconductors were deposited on the cleaned SiO_2_/Si substrate by sputter. For single IGZO TFTs, IGZO (In: Ga: Zn:O = 1:1:1:4 at %) was deposited using a radio‐frequency (RF) magnetron sputtering system with 90 W of sputtering power, Ar gas (25 sccm) as working gas, and a working pressure of 10 mTorr. For the bottom layer of dual‐layer TFTs, the ITO target (In_2_O_3_:SnO_2_ = 9:1 wt.%) was deposited using a direct‐current (DC) magnetron sputtering system co‐sputtered with the IGZO. Co‐sputtering with the ITO target and IGZO target was conducted at 27 W and 90 W of sputtering power, respectively. IGZO was solely deposited for the top layer, same conditions as single IGZO TFTs. After sputtering the OS layer(s), the layers were annealed at 250 °C for 90 s using the rapid thermal annealing (RTA) method under atmospheric conditions. Aluminum (100 nm) was deposited on the OS layer by thermal evaporation using metal shadow masks defining the dimension of channel width and length as 1000 and 50 µm, respectively. Using a photomask, the photolithography and wet‐etching process were executed to avoid excessive leakage current.

### Measurement and Analysis

The chemical composition of OS layers was analyzed using X‐ray photoelectron spectroscopy (XPS) (AXIS SUPRA, Kratos) with a monochromic Al *a*K X‐ray source. The thickness of all layers was measured with an Alpha‐Step profiler. The current–voltage characteristics of all OS TFTs were measured using an Agilent 4155B semiconductor parameter analyzer with a compliance current of 10^−2^ A at room temperature in dark conditions. The transfer curves of all TFTs were measured by sweeping the gate voltage (*V*_GS_) from −70 to +70 V, with a voltage between the drain and source (V_DS_) of +20 V. Stability of positive bias stress (PBS) was measured over 3600 s at +20 V of V_GS_ and +10 V of V_DS_. The stability of negative bias stress (NBS) was measured under the same conditions as PBS, except for the V_DS_ with −10 V.

### Machine Learning Modeling and Bayesian Optimization

Bayesian optimization (BO) was conducted by establishing Gaussian process regression (GPR) and using expected improvement (EI) as an acquisition function for the decision of the most valuable subsequential experiment. This model was based on the library in the scikit‐learn 1.2.2 package.^[^ ^39^ ^]^

The black‐box function including the thickness of OS layers (*x*, input data) and electrical performances (*y*, output data) was represented as ***f*** (***x***): thickness of the bottom layer (***x***_***i*** 1_), thickness of the top layer (***x***_***i*** 2_), field effect mobility (***y***_***i*** 1_), and *V*_th_ difference related to a single IGZO TFT (***y***_**i**2_). The subscript *i* denotes the number of experiments. These samples are incorporated into the data set ***D *** = (***x***, ***y***). GPR was utilized to make predictions for a new experimental outcome, ***f*** (x_*_), given a specific set of conditions, x_*_. The optimal conditions were selected from a range of possible function distributions. Assume that *f(x)* follows multivariate Gaussian distribution, it can be expressed as follows using the mean function ***μ*** (***x***), and the kernel (covariance) function ***K*** (***x***_***i***_, ***x***_**j**_) through the data set *D*.

(3) $$f\left(x\right)∼\mathrm{GP}\left(\mu \left(x\right),K\left(x_{i},x_{j}\right)\right)$$

(4) $$\left[\begin{array}{c} f\left(x\right)\\ f\left(x_{*}\right) \end{array}\right]∼N\left(\left[\begin{array}{c} \mu \left(x\right)\\ \mu \left(x_{*}\right) \end{array}\right],\left[\begin{array}{cc} K\left(x,x\right) & K\left(x,x_{*}\right)\\ K\left(x_{*},x\right) & K\left(x_{*},x_{*}\right) \end{array}\right]\right)$$

The GPR model was computed to predict posterior distribution (x_*_,  y_*_) based on the observed values. In GPR, the kernel function determines the covariance between any two points in the input space, and the hyperparameter of the kernel controls its shape and scale. The kernel equation and its hyperparameters were determined by the type and characteristics of the data being analyzed.

Applying Bayes rule with the conditional probability distribution, (x_*_,  y_*_) was modeled through a multivariate Gaussian distribution based on the kernel function (*K*) as:

(5) $$\mu_{y_{*}|y}=\mu_{*}+K\left(x_{*},x\right){\left[K\left(x,x\right)+\sigma^{2}l\right]}^{-1}\left(y-\mu \left(x\right)\right)$$

(6) $$\sigma_{y_{*}|y}=K\left(x_{*},x_{*}\right)-K\left(x_{*},x\right){\left[K\left(x,x\right)+\sigma^{2}l\right]}^{-1}K\left(x,x_{*}\right)$$

Here, *l* is the length‐scale hyperparameter of the kernel function. Even if only *l* was explicitly included as separate terms, all hyperparameters are implicitly included in the kernel matrix (*K*).

When dealing with one input data (thickness of the bottom layer) and one output data (field effect mobility), the radial basis function (RBF) was used as the kernel function.

(7) $$K\;\left(x_{i},x_{j}\right)=\exp\left(-\frac{d{\left(x_{i},x_{j}\right)}^{2}}{2l^{2}}\right)$$

where ***d*** (***x***_**i**_, ***x***_**j**_) is the Euclidean distance between *x*_i_ and *x*_j_, and *l* is the length‐scale parameter. It means that the RBF kernel calculates the covariance between data solely on their distance from each other.

When input and output data to be calculated increased from 1 to 2, the RBF kernel was not the proper kernel for capturing the complex relationships between data. Furthermore, the complexity was further intensified as the tendency for the electrical performance of the bottom layer and the top layer is different (anisotropic). The rational quadratic (RQ) kernel is a more flexible function using 2 hyperparameters: length‐scale parameter *l* and scale‐mixture parameter *a*.

(8) $$K\;\left(x_{i},x_{j}\right)={\left(1+\frac{d{\left(x_{i},x_{j}\right)}^{2}}{2\alpha l^{2}}\right)}^{-\alpha }$$

Optimal hyperparameters were determined by maximizing the logarithm of the marginal likelihood (LML).

EI is a commonly used acquisition function in BO algorithms. It was calculated based on a surrogate model (GPR) to estimate the potential improvement of a candidate solution over the current best solution. EI was designed to balance the trade‐off between exploration and exploitation methods. Exploration involved searching the region of highest uncertainty, while exploitation focused on the region where the ***f*** (x_*_) was maximized. The equation for EI based on GPR is:

(9) $$\mathrm{EI}\;\left(x_{*}\right)=\left(\mu_{*}\left(x_{*}\right)-f\left(x^{+}\right)\right)\;\Phi \left(Z\right)+\sigma_{*}\left(x_{*}\right)\phi \left(Z\right)$$

(10) $$Z=\frac{\mu_{*}\left(x_{*}\right)-f\left(x^{+}\right)}{\sigma_{*}\left(x_{*}\right)}$$

Here, **Φ** and ϕ are the cumulative distribution function and probability density function of the standard normal distribution, and ***f*** (***x*** ^+^) is the current best‐observed function value.

The entire design procedure for engineering the dual‐layer TFTs, including hyperparameter tuning, training, and EI optimization, was completed in under 90 s on a MacBook with an M1 chip.

### Statistical Analysis

*Preprocessing of Data*: The performances of all devices were measured on the same equations and standards. The field effect mobility was calculated utilizing the linear mobility equation as follows:

(11) $$I_{D}=\mu C_{i}\frac{W}{L}\left(\left(V_{GS}-V_{th}\right)V_{DS}-\frac{V_{DS}^{2}}{2}\right)$$

where ***μ*** is the field effect mobility, ***C***_**i**_ is the capacitance of the gate insulator, W is the channel width, L is the channel length, ***V***_**GS**_ is the gate voltage, ***V***_**th**_ is the threshold voltage, and ***V***_**DS**_ is the draining voltage.

The subthreshold swing was calculated as follows:

(12) $$SS={\left({\left.\frac{dlog\left(I_{DS}\right)}{dV_{GS}}\right|}_{max}\right)}^{-1}$$

The threshold voltage was derived by interpolating the point where the drain current of the transfer curve first exceeds 1 nA and the point before it in the curve.

*Data Presentation and Sample Size*: For Bayesian optimization with respect to a single variable, a total of nine input variables were set (sample size *n* = 9), ranging from 0 to 80 s at intervals of 10 s. Among these, seven data (*n*_1_ = 7) were ultimately selected for training. For two input variables, a set of 81 input variables was set (sample size *n* = 81), and finally, 15 data points (*n*_2_ = 15) from this set were chosen for training.

Statistical methods used to assess significant differences with sufficient details: All performance parameters were measured at various points of the devices fabricated under the same conditions to calculate the median and deviation. For *n* samples, the median and standard deviation were obtained using the following method and equation.

Median: ${\left(\frac{n+1}{2}\right)}^{\mathrm{th}}$ observation (n is odd), and $\frac{{\left(\frac{n}{2}\right)}^{\mathrm{th}}+{\left(\frac{n}{2}+1\right)}^{\mathrm{th}}}{2}$ observation (n is even)

(13) $$\mathrm{Standard}\;\mathrm{deviation}:\;\sigma =\sqrt{\frac{\sum {\left(x_{i}-\mu \right)}^{2}}{n}}$$

where *n* is the sample number and ***μ*** is the average value.

*Software used for Statistical Analysis*: The compilation was conducted in the Python 3.11 environment using the Spyder application. The scikit‐learn module is called for the algorithm library.

## Conflict of Interest

The authors declare no conflicts of interest.

## Supporting information

Supporting Information

Supplemental Movie 1

## Data Availability

The data that support the findings of this study are available from the corresponding author upon reasonable request.
