# Supplementary material for: Machine Learning Driven Channel Thickness Optimization in Dual‐Layer Oxide Thin‐Film Transistors for Advanced Electrical Performance
Source: Adv Sci (Weinh). 2023 Nov 20;10(36):2303589. doi: 10.1002/advs.202303589 (PMC10754089; doi:10.1002/advs.202303589)
Supplement: Supplementary file 1 — Supporting Information [file ADVS-10-2303589-s001.pdf]

ADVANCED SCIENCE
Open Access

Supporting Information

for *Adv. Sci.*, DOI 10.1002/advs.202303589

Machine Learning Driven Channel Thickness Optimization in Dual-Layer Oxide Thin-Film Transistors for Advanced Electrical Performance

Jiho Lee, Jae Hak Lee, Chan Lee, Haeyeon Lee, Minho Jin, Jiyeon Kim, Jong Chan Shin, Eungkyu Lee and Youn Sang Kim**

Supporting Information

Machine Learning Driven Channel Thickness Optimization in Dual-Layer Oxide Thin-Film Transistors for Advanced Electrical Performance

Jiho Lee, Jae Hak Lee, Chan Lee, Haeyeon Lee, Minho Jin, Jiyeon Kim, Jong Chan Shin, Eungkyu Lee, and Youn Sang Kim**

This supplementary material includes:

Figure S1. Hysteresis characteristics of pristine IGZO, CST-10 s, 30 s and 50 s TFTs

Figure S2. Transfer curves of single IGZO TFTs and dual (CST-90 s) layer TFTs

Figure S3. Single IGZO film and co-sputtering (ITO and IGZO) film thickness distribution

Figure S4. Experimental verification of the predicted values. The sputtering time for each bottom and top layer set at (40 s, 650 s) for the blue line and (60 s, 670 s) for the red line

Table S1. Details of experimental conditions

Table S2. Details of the 95 % confidence interval for the predicted values and measured values of mobility and threshold voltage at (40 s, 650 s) and (60 s, 670 s)

Supporting Note 1. Effect of bottom layer thickness on the electrical characteristics of dual-layer TFTs.

Supporting Note 2. Detailed equations for Figure of Merit (FoM)

Movie S1. Entire work process of BO about bottom layer and field effect mobility

Supporting references.

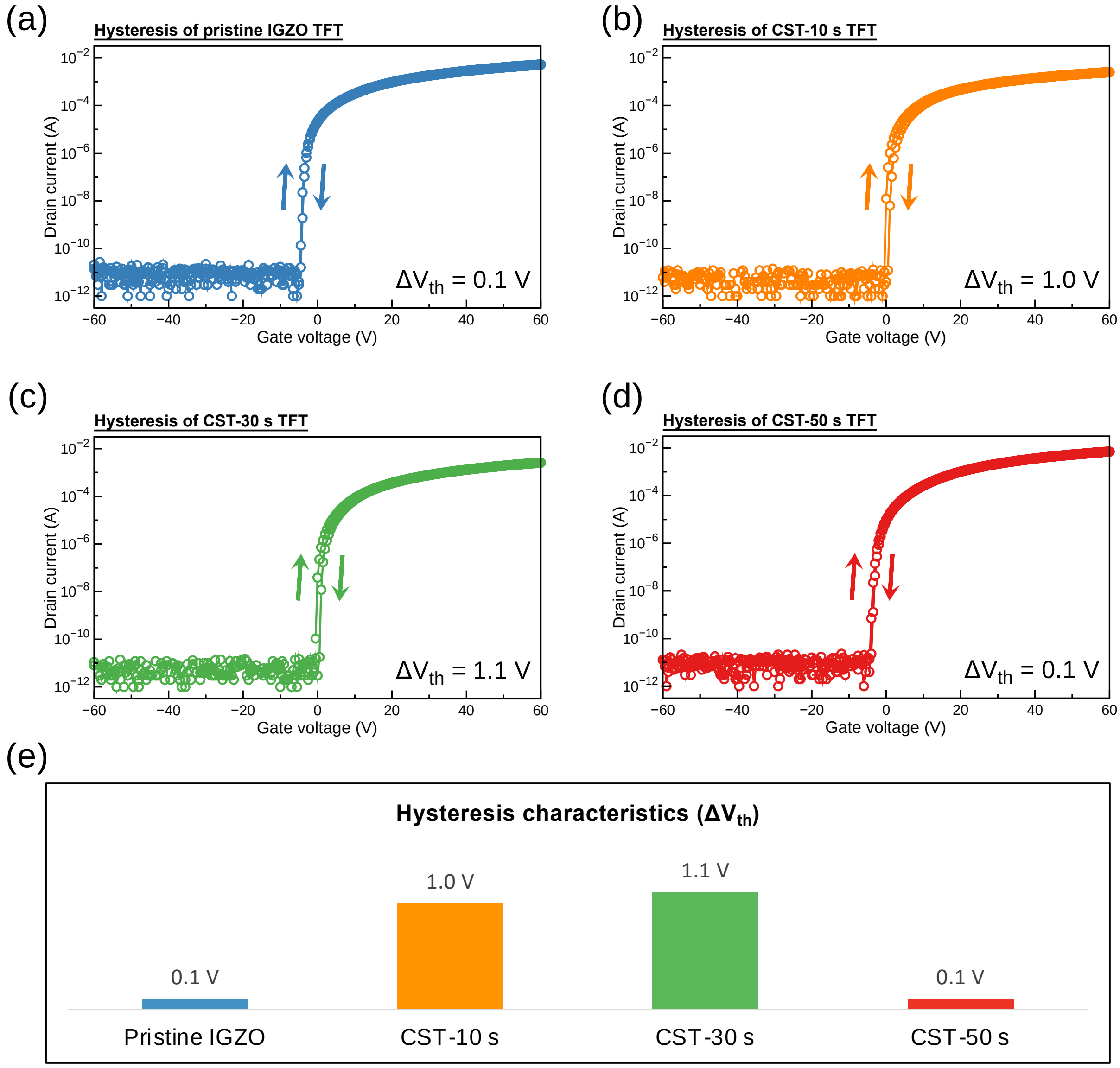

Figure S1. Hysteresis characteristics of pristine IGZO, CST-10 s, 30 s and 50 s TFTs (a) hysteresis of pristine IGZO TFT (b) CST-10 s TFT (c) CST-30 s TFT (d) CST-50 s TFT (e) V_{th} shift (hysteresis characteristics) of pristine IGZO, CST-10s, 30 s and 50s TFTs.

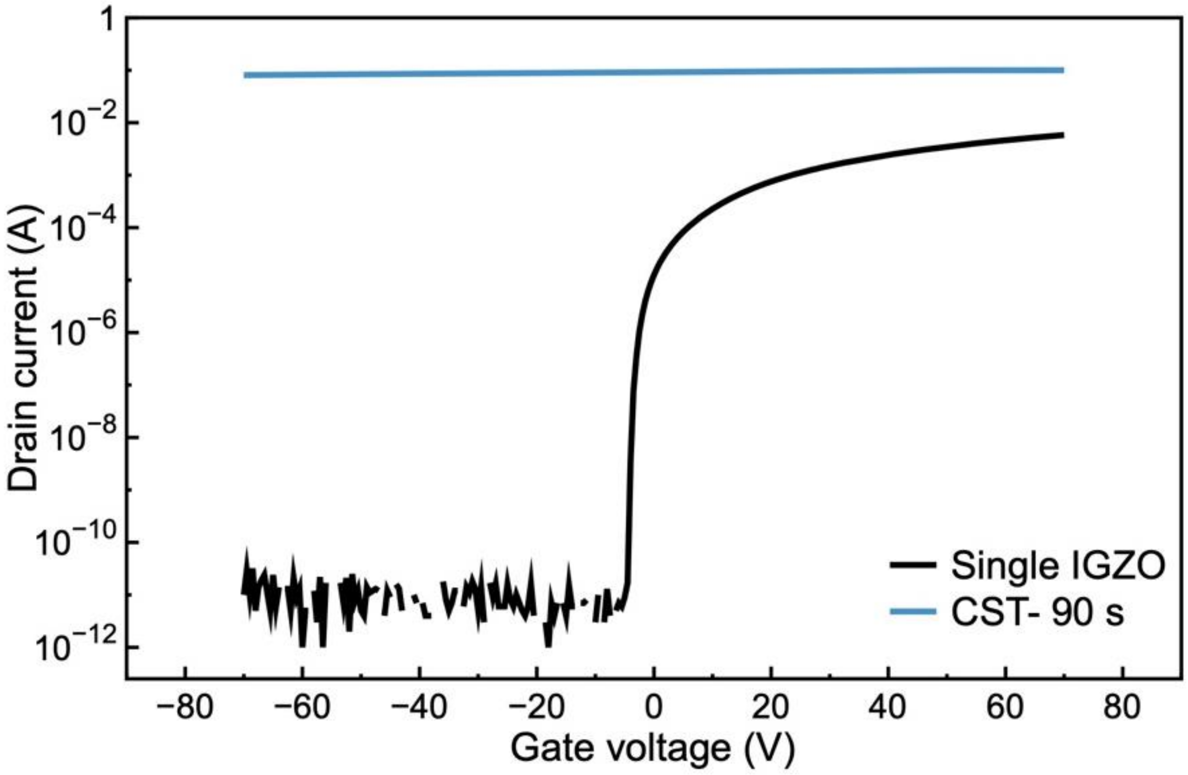

Figure S2. Transfer curves of single IGZO TFTs and dual (CST-90 s) layer TFTs.

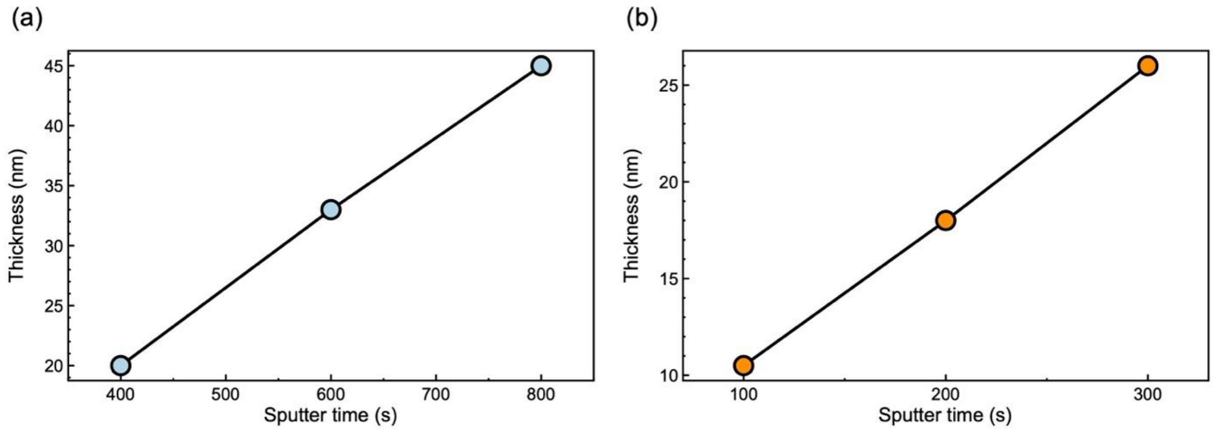

Figure S3. Thickness distribution according to sputter time where (a) displays single IGZO film and (b) shows co-sputter (ITO and IGZO) films.

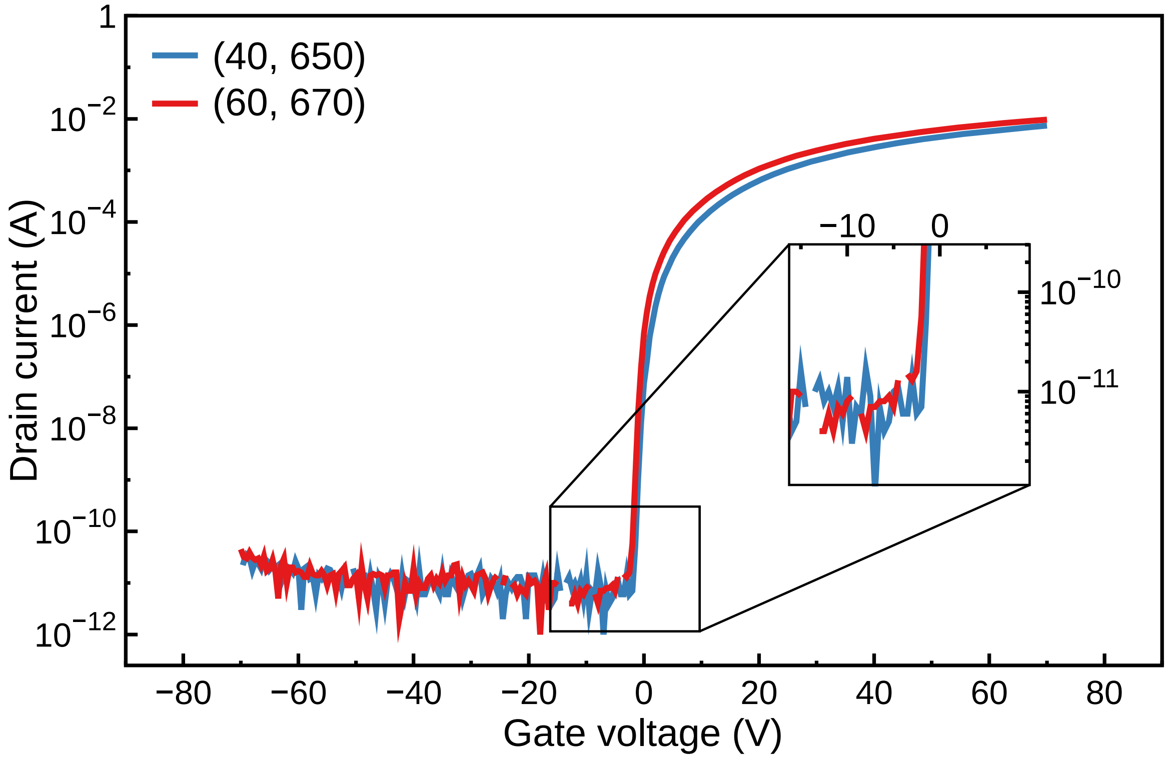

Figure S4. Experimental verification of the predicted values. The sputtering time for each bottom and top layer set at (40 s, 650 s) for the blue line and (60 s, 670 s) for the red line.

Input (Co-sputtering time (s))		Output	
Bottom layer	Top layer	μ (cm^2 V^{-1} s^{-1})	V_{th} (V)
0	600	18.2 ± 1.02	-3.02 ± 0.18
0	640	17.6 ± 1.25	-3.5 ± 0.23
0	680	18.4 ± 0.28	-3.6 ± 0.23

10	670	15.6 ± 0.74	-0.6 ± 0.33
20	660	19.4 ± 0.85	-2.1 ± 0.31
30	650	20.3 ± 0.29	-1.6 ± 0.29
40	640	29.7 ± 0.24	-2.5 ± 0.49
50	600	28.9 ± 0.80	-4.5 ± 0.37
50	630	36 ±1.15	-3.5 ± 0.45
50	680	28.4 ± 0.77	-2.5 ± 0.24
60	620	31.2 ± 0.51	-5 ± 0.38
70	610	31.8 ± 0.25	-6.5 ± 0.41
80	600	31.5 ± 0.11	-5.6 ± 0.34
80	640	33.4 ± 2.85	-4.6 ± 0.37
80	680	30.3 ± 1.84	-4 ±0.47

Table S1. Details of experimental conditions

**Input (Co-sputtering time (s))**		**Output**		**Measured values**	
Bottom layer	Top layer	μ ($cm^2 V^{-1} s^{-1}$)	V_{th} (V)	μ ($cm^2 V^{-1} s^{-1}$)	V_{th} (V)
40	650	25.0 ~ 26.5	-3.48 ~ -0.91	25.7 ± 0.25	-1.02 ± 0.40

60	670	28.1 ~ 30.2	-4.71 ~ -1.42	30.0 ± 0.80	-1.51 ± 0.30

Table S2. Details of the 95 % confidence interval for the predicted values and measured values of mobility and threshold voltage at (40 s, 650 s) and (60 s, 670 s).

Supporting Note 1. Effect of bottom layer thickness on the electrical characteristics of dual-layer TFTs.

The electrical performance of the device in this study is affected by two structural characteristics: In a dual-layer structure, the bottom layer plays a crucial role as the active channel where electrons accumulate due to gate bias [S1]. A very thin layer is used for the bottom layer, and the characteristics of oxide semiconductors are thickness-dependent. As the thickness of the film decreases, oxide semiconductors deposited via sputtering systems exhibit an increase in interface traps, acceptor like tail states and subgap DOS (density-of-state), which originate from structural disorder and defects [S2-4]. This leads to V_{th} shift and stability deterioration due to charge trapping. A higher trap density results in positive V_{th} shift in single sweep transfer curve because a higher gate bias is required to obtain the same channel carrier density, overcoming the occupancy of the traps. And the presence of clockwise hysteresis in transfer curves is consistent with electron trapping at/near the interface between the gate oxide and active layer [S5]. The hysteresis window signifies trapped electrons or mobile charges [S6]. Not only does the total trap density influence stability test, including hysteresis and bis stress such as PBS and NBS, but oxygen vacancy (V_O) also does, because V_O act as both shallow donors and carrier traps [S7]. It's worth noting that the band dissociation energy of Sn-O is 528 kJ/mol which is notably higher than In-O (346 kJ/mol), Ga-O (374 kJ/mol) and Zn-O (248 kJ/mol), and thus, the introduction of Sn atoms through the co-sputtering of ITO and IGZO suppresses the formation of V_O [S8].

In summary, as the thickness of the bottom layer increases with increased ITO co-sputtering time, there is a corresponding decrease in the total trap density. These defects serve as charge traps under bias stress, affecting carrier concentration and conduction pathways. Consequently, improved conduction pathway with reduced trap density leads to an appropriate V_{th} and improved stability.

Supporting Note 2. Detailed equations for Figure of Merit (FoM)

In this section, we explain the specific procedure to derive the Figure of Merit (FoM) as described in section 2.3 of manuscript. As show in Equation 2, both field effect mobility and V_{th} are normalized for having value between 0 and 1. When field effect mobility calculated using Equation 1, it indicates 1 when it is the maximum value and 0 at the minimum value. This normalized field effect mobility means *m* of equation 2. As mentioned before, a higher field effect mobility signifies improved performance, whereas a smaller absolute value of V_{th}, closer to 0, represents a more desirable electrical performance with respect to power consumption. In order to reflect these aspects, the normalization of V_{th} initially sets 0 as the minimum value (x_{min}).

$$(V_{th})_{normalized} = \frac{|V_{th}|}{|V_{th}|_{max}} \tag{S1}$$

The normalized V_{th}, determined through this calculation, approaches a value of 0 as absolute value of V_{th} gets closer to 0. To ensure that both the field effect mobility and V_{th} term within the FoM (Equation 2) approach a value close to 1 as they exhibit better electrical performance, the V_{th} term is calculated as follows:

$$t^* = 1 - (V_{th})_{normalized} \tag{S2}$$

Re-normalization is performed to achieve a normalized value between 0 and 1.

$$t_{normalized} = \frac{t^* - t^*_{min}}{t^*_{max} - t^*_{min}} \tag{S3}$$

Equation 2 is calculated by substituting the normalized mobility (*m*) and V_{th} (*t*) through this process.

Supporting references

[S1] C. Im, J. Kim, N. K. Cho, J. Park, E. G. Lee, S. E. Lee, H. J. Na, Y. J. Gong, Y. S. Kim, *ACS Appl Mater Interfaces* **2021**, 13, 51266.

[S2] M. J. Kim, H. J. Park, S. Yoo, M. H. Cho, J. K. Jeong, *IEEE Transactions on Electron Devices* **2022**, 69, 2409.

[S3] S. Y. Lee, D. H. Kim, E. Chong, Y. W. Jeon, D. H. Kim, *Applied Physics Letters* **2011**, 98.

[S4] J. X. Jiang, T. Matsuda, M. Kimura, S. Y. Liu, M. Furuta, *Journal of Nano Research* **2017**, 46, 93.

[S5] Q. Li, J. Dong, D. Han, Y. Wang, *Membranes (Basel)* **2021**, 11, 929.

[S6] K. Liang, D. Li, H. Ren, M. Zhao, H. Wang, M. Ding, G. Xu, X. Zhao, S. Long, S. Zhu, P. Sheng, W. Li, X. Lin, B. Zhu, *Nanomicro Lett* **2021**, 13, 164.

[S7] H. K. Noh, K. J. Chang, B. Ryu, W. J. Lee, *Physical Review B* **2011**, 84, 115205.

[S8] N. Saito, T. Ueda, T. Tezuka, K. Ikeda, *IEEE Journal of the Electron Devices Society* **2018**, 6, 1253.
